# MeTime: an R package for reproducible longitudinal metabolomics data analysis

**DOI:** 10.1093/bioadv/vbag252

**Published:** 2026-08-31

**Authors:** Bharadwaj Marella, Patrick Weinisch, Lara Vehovec, Vinh Tran, Josef J Bless, Yacoub A Njipouombe Nsangou, Gabi Kastenmüller, Matthias Arnold

**Affiliations:** Institute of Computational Biology, Helmholtz Zentrum München, German Research Center for Environmental Health, Neuherberg, Germany; Institute of Computational Biology, Helmholtz Zentrum München, German Research Center for Environmental Health, Neuherberg, Germany; Institute of Computational Biology, Helmholtz Zentrum München, German Research Center for Environmental Health, Neuherberg, Germany; Institute of Computational Biology, Helmholtz Zentrum München, German Research Center for Environmental Health, Neuherberg, Germany; Institute of Computational Biology, Helmholtz Zentrum München, German Research Center for Environmental Health, Neuherberg, Germany; Institute of Computational Biology, Helmholtz Zentrum München, German Research Center for Environmental Health, Neuherberg, Germany; Institute of Computational Biology, Helmholtz Zentrum München, German Research Center for Environmental Health, Neuherberg, Germany; Institute of Computational Biology, Helmholtz Zentrum München, German Research Center for Environmental Health, Neuherberg, Germany; Department of Psychiatry and Behavioral Sciences, Duke University, Durham, NC, USA

## Abstract

**Summary:**

MeTime is an opensource R package for reproducible analysis of longitudinal metabolomics data. It builds upon a central S4 container, metime_analyser, that stores multiple datasets, associated metadata and analysis outputs, enabling unified handling of complex longitudinal studies. Analyses are constructed by piping modular functions, beginning with data transformations (mod_*), followed by calculations (calc_*), and optional meta-analysis (meta_*), so entire workflows remain transparent and easy to modify. MeTime wraps numerous existing methods within a consistent interface, including sample and metabolite distributions, correlation/distance matrices, dimensionality reduction (PCA, UMAP, t-SNE), random forest imputation and feature selection via Boruta, eigenmetabolites and WGCNA-based clustering, conservation index analysis, regression models (linear, mixed-effects, and generalized additive), and partial-correlation networks. By retaining all intermediate results and provenance within the container, MeTime facilitates iterative exploration and ensures reproducible reporting via automatically generated HTML/PDF outputs. Comprehensive user guides, case studies and reference documentation accompany the package, making MeTime a versatile platform for longitudinal omics workflows.

**Availability and implementation:**

MeTime is available as an open-source R package and can be installed directly from GitHub (https://github.com/compneurobio/MeTime). Source code, installation instructions, documentation, tutorials, and reproducible case-study workflows are provided within the repository. MeTime has been tested on Microsoft Windows, Unix/Linux, and macOS. For users requiring a containerized environment, a Docker implementation is additionally provided through the GitHub Container Registry.

## 1 Introduction

Longitudinal metabolomics provides a powerful framework for characterizing temporal changes in metabolite profiles within an organism. By capturing within-subject dynamics over time, longitudinal designs enable the investigation of molecular responses to stimuli, interventions, and disease progression, while accounting for inter-individual variability. Metabolomic profiles provide a downstream readout of biochemical variation shaped by molecular, environmental, lifestyle, and disease-related influences. Longitudinal measurements can capture dynamic changes associated with disease progression, intervention response, and predict future health outcomes, including all-cause mortality (Lacruz *et al.* 2018, Rosato *et al.* 2018, Arnold *et al.* 2020, Castelli *et al.* 2022, Shen *et al.* 2023).

Advances in mass spectrometry technologies and data acquisition workflows have led to a rapid increase in the generation of longitudinal metabolomics datasets. This growth has intensified the demand for scalable, flexible, and reproducible computational approaches capable of handling high-dimensional, multi-timepoint data (Wishart *et al.* 2022, Trifonova *et al.* 2023). The analysis of such data commonly relies on a combination of multivariate methods, regression-based approaches (Gibbons *et al.* 2010), conservation index analysis (Yousri *et al.* 2014), and network-based models to capture temporal structure and inter-metabolite relationships (Krumsiek *et al.* 2011, Epskamp *et al.* 2018).

In parallel, the metabolomics community has placed increasing emphasis on reproducibility, transparency, and open science practices (Considine *et al.* 2017, Du *et al.* 2022). Contemporary publication standards often require the sharing of source code, parameters, and intermediate results alongside manuscripts (Baker 2016). While these practices improve scientific rigor, they also impose a substantial burden on researchers, who must design analysis pipelines that are both flexible and fully traceable.

Several toolboxes support metabolomics data preprocessing, statistical analysis, visualization or pathway interpretation, such as MetaboAnalyst, maplet, and MeTEor (Chong *et al.* 2018, Chetnik *et al.* 2022, Grabert *et al.* 2024); however, these tools were generally not designed around the specific requirements of longitudinal metabolomics studies involving repeated measurements, multiple datasets, heterogeneous platforms and the need to preserve analysis provenance across sequential workflows (Supplementary Table 1, available at supplementary material  *Bioinformatics Advances* online). Here, we present MeTime, an open-source R package (https://github.com/compneurobio/MeTime/) that brings together longitudinal metabolomics analyses in a single unified framework: it stores multiple datasets in a central S4 object, wraps a range of existing analysis methods behind a consistent interface, supports modular pipeline construction, and generates self-contained reports for transparent and reproducible workflows and results. In addition to describing the software design and functionality, we demonstrate the use of MeTime through an executable longitudinal metabolomics case study, illustrating how complex analytical pipelines and their results can be generated, stored, and reported in a fully reproducible manner.

## 2 Implementation and functionality

MeTime enables transparent and reproducible analytical pipelines for longitudinal omics data by centering all inputs, outputs, and provenance in a single S4 container. This object stores raw and processed metabolite abundance data from both targeted and untargeted metabolomics platforms, analytical results, and detailed metadata protocoling each pipeline step (called functions, parameter settings, and execution order). Pipelines are assembled by sequentially passing the container through S4 methods using the native R pipe operator (|>) or %>% from magrittr (Bache *et al.* 2022), making analyses straightforward to review, reproduce, and retrace.

**Figure 1:**
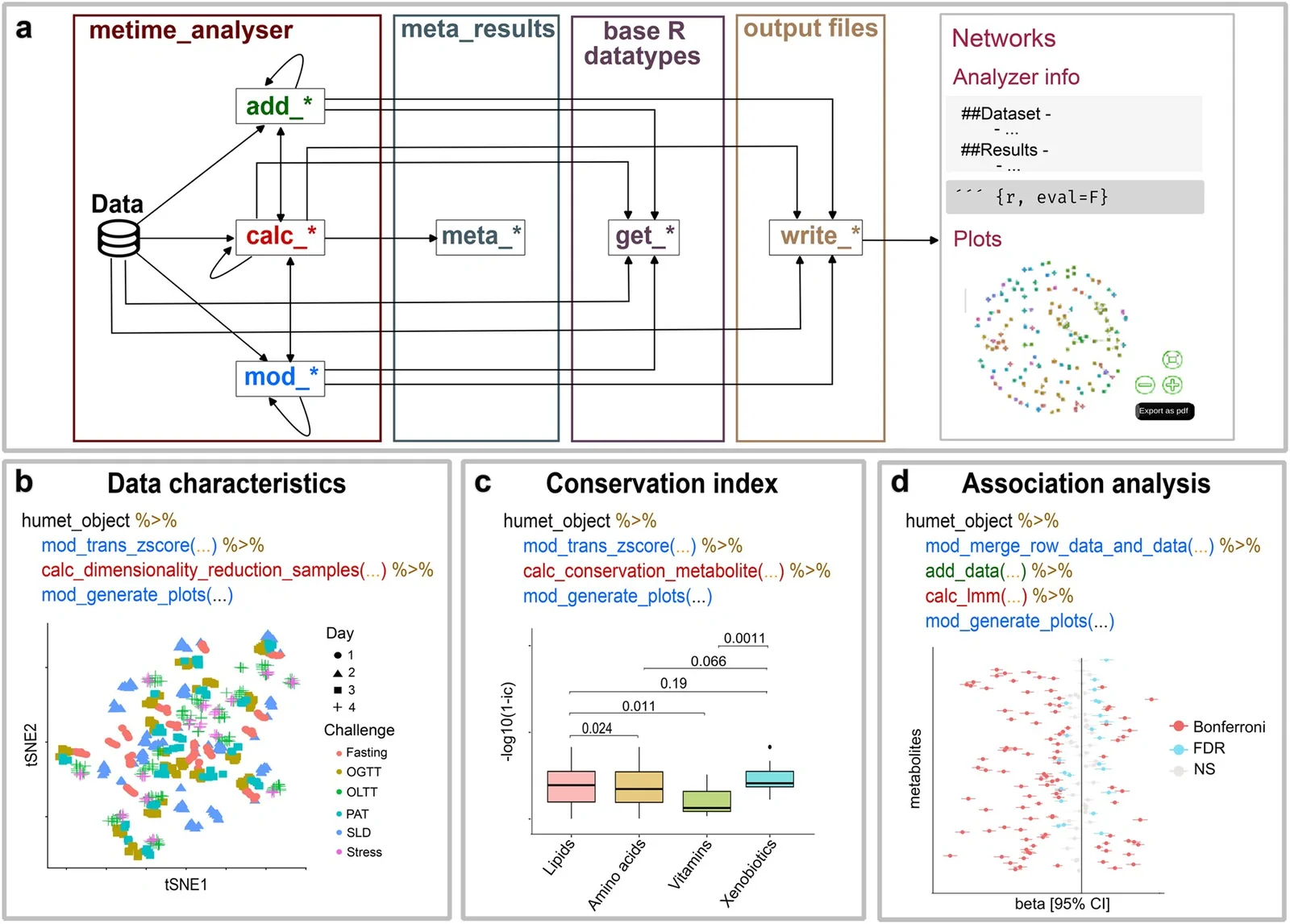
Overview of MeTime’s architecture and example analyses. (a) A schematic of constructing a pipeline using MeTime: the central metime_analyser object holds input data and results; functions prefixed as add_* import or merge data, mod_* modify and annotate, calc_* perform analyses, meta_* compare summary statistics, get_* retrieves contents, and write_* generates reports. Arrows indicate, which function family can be applied consecutively. Summary statistics across two calculations or metime_analyser objects can be summarised in secondary meta_results objects and visualised via built-in plotting mechanisms. The remaining panels showcase examples on how the user can build such pipelines. (b) Data characteristics: an example pipeline that *z*-scores metabolite abundances, computes a *t*-SNE embedding, and plots samples by challenge and day of challenge. (c) Conservation index: box plots showing stability of metabolic profiles across lipid and non-lipid classes after transformation and conservation index calculation. (d) Association analysis: effect-size estimates and 95% confidence intervals for selected metabolites from a linear mixed-effects model; colors indicate significance after Bonferroni, FDR adjustment (*q* < 0.05) and nominal (*P* < 0.05) thresholds.

A key design goal is native support for longitudinal metabolomics projects that include multiple datasets (e.g. cohorts, experimental batches, or analytical platforms) without requiring full sample overlap. While existing container classes such as SummarizedExperiment (Morgan *et al.* 2023) and data structures implemented in maplet (Chetnik *et al.* 2022) provide robust solutions for single datasets, they do not directly address coordinated workflows spanning multiple heterogeneous datasets. MeTime addresses this by enabling independent yet synchronized analyses across datasets while storing all derived outputs within the data container (Fig. 1). Meta-analysis summaries are stored separately in a second dedicated class object, which cleanly separates primary results within one container or across two containers from downstream meta-analysis. See Supplementary Information, available at supplementary material  *Bioinformatics Advances* online for more details on the architecture of these classes.

MeTime follows a modular philosophy in which each analytical component (e.g. feature selection, regressions, data driven networks) is implemented as an independent function that can be combined into complete workflows. Functions follow a standardized tagging scheme reflecting their role in the pipeline (Fig. 1), enabling users to insert, remove, or reorder steps with minimal effort and support rapid prototyping. The package currently comprises ∼80 functions spanning data loading, extraction, modification, augmentation, statistical analysis, meta-analysis of summary statistics, and export. Statistical methods include missing value imputation, dimensionality reduction, linear models, linear mixed-effects models, generalized additive mixed models, conservation index analysis, and data driven networks, alongside utilities for publication-ready visualizations and structured result tables. Although developed for metabolomics, the underlying architecture is extensible to other omics modalities. Templates and extensive documentation are provided to facilitate extension with custom functions while preserving compatibility with the core architecture (https://github.com/compneurobio/MeTime/).

Following pipeline execution, results remain embedded in the data container with the option to generate comprehensive PDF or HTML reports via knitr and Rmarkdown packages (Allaire *et al.* 2023). Reports enumerate pipeline steps in the order executed, including function names, parameter settings, and associated tables/figures (Fig. 1), and can be exported for sharing and archival. By consolidating results with code provenance and parameterization in a single document, MeTime supports transparent reporting and reproducibility.

## 3 Exemplary analysis

To demonstrate the functionality of MeTime, we provide two complementary usage examples. First, the package includes the publicly available HuMet dataset (Weinisch *et al.* 2024), which is used in the GitHub tutorials and example workflows as a small and accessible dataset for learning the package and exploring its main functions along with a complete walkthrough of the different analyses including dimensionality reduction, feature selection, conservation index analysis, association analysis, network analysis and cluster identification that can be conducted using MeTime in a dedicated Rmarkdown file (Supplementary Information, available at supplementary material  *Bioinformatics Advances* online). Second, we provide a fully reproducible workflow on GitHub (https://github.com/compneurobio/MeTime/tree/main/docs/case-studies) that replicates the association analyses using a controlled-access longitudinal metabolomics dataset from the Alzheimer’s Disease Neuroimaging Initiative (ADNI) measured with the targeted Biocrates MXP® Quant 500 platform from Marella *et al.* 2025. After quality control and construction of the data container, this analysis can be rerun directly using the provided code. The analyses resulting from Supplementary Infromation are provided in the Supplementary Data, available at supplementary material  *Bioinformatics Advances* online as report files, so that each code snippet can be viewed both as code and as its resulting output. Together, these examples show that MeTime can be used both as an easy starting point for new users and as a reproducible framework for a wide range of longitudinal omics analyses.

## 4 Limitations

MeTime is not intended to replace available tools such as maplet but rather to complement and add missing components for longitudinal data analysis. Therefore, current limitations include limited preprocessing functionality beyond export/import functions to interface with these tools. Furthermore, some analyses as implemented in MeTime are computationally intensive. Although we provide support for a popular high-performance computing environment (SLURM), integration with workflow managers such as targets is currently not included but will be provided in future releases. Finally, while the most commonly used longitudinal metabolomics analysis methods are available in MeTime, it does not cover all possible statistical methods. However, the framework is modular and extensible, with developer documentation provided for users who wish to add methods for their own applications.

## 5 Conclusion

MeTime provides a comprehensive framework for the reproducible analysis of longitudinal metabolomics data by enabling rapid construction of transparent and modular analytical pipelines. By integrating data management, statistical modelling, results storage, and report generation within an S4-based architecture, MeTime thereby reduces the technical overhead associated with longitudinal data analysis, supporting both methodological development and transparent scientific reporting.

## Supplementary Material

vbag252_Supplementary_Data

## Data Availability

No new primary data were generated in this study. The HuMet dataset used for tutorials and example analyses is publicly available through the HuMet Repository (https://www.humet.org) and is distributed with MeTime for demonstration purposes. Additional sample information and spectral data are available through MetaboLights under accession MTBLS89. The Alzheimer's Disease Neuroimaging Initiative (ADNI) longitudinal metabolomics data used in supplementary case studies is available through the AD Knowledge Portal (doi:10.7303/9618123), associated clinical and demographic data is available through the ADNI data-sharing platform at LONI. The MeTime source code, documentation, case study presented here, and reproducible analysis workflows are openly available at https://github.com/compneurobio/MeTime/. Scripts and corresponding reports underlying the examples presented in this study are provided as Supplementary Data.
