# Supplementary material for: MeTime: an R package for reproducible longitudinal metabolomics data analysis
Source: Bioinform Adv. 2026 Aug 31;6(1):vbag252. doi: 10.1093/bioadv/vbag252 (PMC13568824; doi:10.1093/bioadv/vbag252)
Supplement: Supplementary file 1 [file vbag252_supplementary_data.zip › dim_red.html]

dim\_red


Code 

- Show All Code
- Hide All Code

# dim\_red

## dim\_red

- UMAP\_samples
  - Analyzer
    info
  - Plots
- tSNE\_samples
  - Analyzer
    info
  - Plots
- Session
  info

2026-08-01

# UMAP\_samples

## Analyzer info

```
## Datasets: 
##  - Dataset: humet_data with 833 samples and 2672 metabolites
##  - Dataset: humet_subset_data with 833 samples and 312 metabolites
## Results: 
## 1.UMAP_samples : 
##  -  dimensionality reduction method: UMAP for samples data of humet_subset_data
## 2.tSNE_samples : 
##  -  dimensionality reduction method: tSNE for samples data of humet_subset_data
```

object %>%   
Copy to Clipboard

mod\_trans\_zscore(which\_data = 'humet\_subset\_data') %>%   
 mod\_mutate(which\_data = 'humet\_subset\_data',type = 'row\_data',mutations = 'Factor.Value.Challenge.Day.=factor, Factor.Value.Challenge.Day.=Factor.Value.Challenge.Day.') %>%   
 calc\_dimensionality\_reduction\_samples(which\_data = 'humet\_subset\_data',type = 'UMAP',cols\_for\_meta = c('Factor.Value.Challenge.', 'Factor.Value.Challenge.Day.'))

×

#### mod\_trans\_zscore

Function to scale the data  
  
Description:  
  
     Modification(mod) Function for scaling datasets  
  
Usage:  
  
       
         (object, which\_data)  
       
Arguments:  
  
  object: An object of class metime\_analyser  
  
which\_data: character vector to define the dataset/s to be used  
  
Value:  
  
     An object of class metime\_analyser with scaled which\_data  
  
See Also:  
  
     base::scale, mod\_trans\_log  
  
Examples:  
  
     # example to apply scaling  
     object <-   
         (object, which\_data=c('dataset1', ...))

Close

×

#### mod\_mutate

Function to mutate columns in row\_data or col\_data  
  
Description:  
  
     Modification(mod) function to mutate data, row\_data, col\_data and  
     results. It is a wrapper function to dplyr::mutate() made for  
     metime\_analyser objects.  
  
Usage:  
  
       
         (object, which\_data, type = 'data', ...)  
       
Arguments:  
  
  object: An S4 object of class metime\_analyser  
  
which\_data: character or numeric input to define Dataset/result of  
          interest. Has to be of length=1  
  
    type: character input of length 1 to define the type of data to be  
          manipulated. Accepted inputs are 'row\_data', 'col\_data',  
          'data' and 'results'. However renamed results will be  
          returned to the user as a list of results and will not return  
          the full analyser object  
  
     ...: arguments to pass directly into dplyr::mutate() function.  
  
Value:  
  
     object with mutated data  
  
See Also:  
  
     mod\_filter, mod\_rename

Close

×

#### calc\_dimensionality\_reduction\_samples

Function to calculate dimensionality reduction methods such as tsne,  
umap and pca.  
  
Description:  
  
     A method to apply on s4 object of class metime\_analyse in order to  
     obtain information after dimensionality reduction on a dataset/s  
  
Usage:  
  
       
         (  
       object,  
       which\_data,  
       type,  
       cols\_for\_meta,  
       stratifications = NULL,  
       name,  
       ...  
     )  
       
Arguments:  
  
  object: An object of class metime\_analyser  
  
which\_data: a character vector - Names of the dataset from which the  
          samples will be extracted  
  
    type: type of the dimensionality reduction method to be applied.  
          Accepted inputs are 'UMAP', 'tSNE', 'PCA'  
  
cols\_for\_meta: A Character vector to define columns names that are to  
          be used for plotting purposes  
  
stratifications: List to stratify data into a subset. Usage  
          list(name=value)  
  
    name: name of the results that you want to have  
  
     ...: additional arguments that can be passed on to prcomp(),  
          M3C::tsne() and umap::umap()  
  
Value:  
  
     a list with two plotter objects containing the dimensionality  
     reduction information that can be parsed into plotting function  
  
Examples:  
  
     #calculate PCA  
     pca <-   
         (object=metime\_analyser\_object,   
      which\_data='name/s of the dataset/s', type='PCA', cols\_for\_meta=c(), stratifications=NULL, name='name of the results')

Close

## Plots

### umap\_samples

dimensionality reduction method: UMAP for samples data of
humet\_subset\_data

# tSNE\_samples

## Analyzer info

```
## Datasets: 
##  - Dataset: humet_data with 833 samples and 2672 metabolites
##  - Dataset: humet_subset_data with 833 samples and 312 metabolites
## Results: 
## 1.UMAP_samples : 
##  -  dimensionality reduction method: UMAP for samples data of humet_subset_data
## 2.tSNE_samples : 
##  -  dimensionality reduction method: tSNE for samples data of humet_subset_data
```

object %>%   
Copy to Clipboard

calc\_dimensionality\_reduction\_samples(which\_data = 'humet\_subset\_data',type = 'tSNE',cols\_for\_meta = c('Factor.Value.Challenge.', 'Factor.Value.Challenge.Day.'))

×

#### calc\_dimensionality\_reduction\_samples

Function to calculate dimensionality reduction methods such as tsne,  
umap and pca.  
  
Description:  
  
     A method to apply on s4 object of class metime\_analyse in order to  
     obtain information after dimensionality reduction on a dataset/s  
  
Usage:  
  
       
         (  
       object,  
       which\_data,  
       type,  
       cols\_for\_meta,  
       stratifications = NULL,  
       name,  
       ...  
     )  
       
Arguments:  
  
  object: An object of class metime\_analyser  
  
which\_data: a character vector - Names of the dataset from which the  
          samples will be extracted  
  
    type: type of the dimensionality reduction method to be applied.  
          Accepted inputs are 'UMAP', 'tSNE', 'PCA'  
  
cols\_for\_meta: A Character vector to define columns names that are to  
          be used for plotting purposes  
  
stratifications: List to stratify data into a subset. Usage  
          list(name=value)  
  
    name: name of the results that you want to have  
  
     ...: additional arguments that can be passed on to prcomp(),  
          M3C::tsne() and umap::umap()  
  
Value:  
  
     a list with two plotter objects containing the dimensionality  
     reduction information that can be parsed into plotting function  
  
Examples:  
  
     #calculate PCA  
     pca <-   
         (object=metime\_analyser\_object,   
      which\_data='name/s of the dataset/s', type='PCA', cols\_for\_meta=c(), stratifications=NULL, name='name of the results')

Close

## Plots

### tsne\_samples

dimensionality reduction method: tSNE for samples data of
humet\_subset\_data

# Session info

```
print(sessionInfo())
```

```
## R version 4.3.2 (2023-10-31 ucrt)
## Platform: x86_64-w64-mingw32/x64 (64-bit)
## Running under: Windows 10 x64 (build 19045)
## 
## Matrix products: default
## 
## 
## locale:
## [1] LC_COLLATE=English_India.utf8  LC_CTYPE=English_India.utf8    LC_MONETARY=English_India.utf8
## [4] LC_NUMERIC=C                   LC_TIME=English_India.utf8    
## 
## time zone: Europe/Berlin
## tzcode source: internal
## 
## attached base packages:
## [1] stats4    stats     graphics  grDevices utils     datasets  methods   base     
## 
## other attached packages:
##  [1] MeTime_1.0.0                lmerTest_3.2-0              lme4_1.1-38                 Matrix_1.6-5               
##  [5] MultiAssayExperiment_1.28.0 maplet_1.1.2                SummarizedExperiment_1.32.0 Biobase_2.62.0             
##  [9] GenomicRanges_1.54.1        GenomeInfoDb_1.38.2         IRanges_2.36.0              S4Vectors_0.40.2           
## [13] BiocGenerics_0.48.1         MatrixGenerics_1.14.0       matrixStats_1.5.0           magrittr_2.0.3             
## [17] lubridate_1.9.4             forcats_1.0.1               stringr_1.6.0               dplyr_1.1.4                
## [21] purrr_1.1.0                 readr_2.1.6                 tidyr_1.3.2                 tibble_3.3.1               
## [25] ggplot2_4.0.1               tidyverse_2.0.0            
## 
## loaded via a namespace (and not attached):
##   [1] fs_1.6.6                bitops_1.0-7            devtools_2.4.5          httr_1.4.7             
##   [5] RColorBrewer_1.1-3      doParallel_1.0.17       Rgraphviz_2.46.0        numDeriv_2016.8-1.1    
##   [9] dynamicTreeCut_1.63-1   profvis_0.3.8           tools_4.3.2             doRNG_1.8.6.2          
##  [13] backports_1.5.0         R6_2.6.1                DT_0.34.0               lazyeval_0.2.2         
##  [17] uwot_0.1.16             mgcv_1.9-1              urlchecker_1.0.1        withr_3.0.2            
##  [21] gridExtra_2.3           tictoc_1.2.1            preprocessCore_1.64.0   fdrtool_1.2.18         
##  [25] textshaping_1.0.4       WGCNA_1.73              cli_3.6.5               logging_0.10-111       
##  [29] network_1.19.0          labeling_0.4.3          sass_0.4.10             KEGGgraph_1.62.0       
##  [33] S7_0.2.1                randomForest_4.7-1.2    systemfonts_1.3.1       bsplus_0.1.5           
##  [37] GeneNet_1.2.17          foreign_0.8-86          longitudinal_1.1.13     sessioninfo_1.2.2      
##  [41] limma_3.58.1            itertools_0.1-3         readxl_1.4.5            rstudioapi_0.18.0      
##  [45] impute_1.76.0           RSQLite_2.3.4           FNN_1.1.4.1             visNetwork_2.1.4       
##  [49] generics_0.1.4          shape_1.4.6.1           crosstalk_1.2.2         car_3.1-3              
##  [53] zip_2.3.0               GO.db_3.18.0            abind_1.4-8             pathview_1.47.1        
##  [57] lifecycle_1.0.5         yaml_2.3.12             carData_3.0-5           SparseArray_1.2.4      
##  [61] Rtsne_0.17              grid_4.3.2              blob_1.3.0              promises_1.2.1         
##  [65] crayon_1.5.3            miniUI_0.1.1.1          lattice_0.22-5          KEGGREST_1.42.0        
##  [69] sna_2.8                 pillar_1.11.1           knitr_1.51              boot_1.3-28.1          
##  [73] corpcor_1.6.10          codetools_0.2-19        glue_1.8.0              data.table_1.18.2.1    
##  [77] remotes_2.4.2.1         vctrs_0.6.5             png_0.1-8               rmdformats_1.0.4       
##  [81] Rdpack_2.6.5            cellranger_1.1.0        gtable_0.3.6            cachem_1.0.8           
##  [85] xfun_0.56               openxlsx_4.2.5.2        rbibutils_2.4.1         S4Arrays_1.2.1         
##  [89] mime_0.12               coda_0.19-4.1           reformulas_0.4.3.1      rsconnect_1.6.1        
##  [93] survival_3.8-3          ggmosaic_0.4.0          pheatmap_1.0.12         iterators_1.0.14       
##  [97] tinytex_0.60            statmod_1.5.0           ellipsis_0.3.2          nlme_3.1-164           
## [101] usethis_2.2.2           bit64_4.6.0-1           bslib_0.10.0            irlba_2.3.5.1          
## [105] otel_0.2.0              rpart_4.1.23            colorspace_2.1-2        DBI_1.2.3              
## [109] Hmisc_5.2-5             nnet_7.3-19             processx_3.8.6          mnormt_2.1.1           
## [113] tidyselect_1.2.1        bit_4.6.0               compiler_4.3.2          MatrixEQTL_2.3         
## [117] glmnet_4.1-10           graph_1.80.0            htmlTable_2.4.3         desc_1.4.3             
## [121] DelayedArray_0.28.0     plotly_4.12.0           bookdown_0.46           checkmate_2.3.3        
## [125] scales_1.4.0            psych_2.3.12            callr_3.7.6             digest_0.6.34          
## [129] minqa_1.2.8             rmarkdown_2.30          XVector_0.42.0          htmltools_0.5.8.1      
## [133] pkgconfig_2.0.3         base64enc_0.1-3         fastmap_1.1.1           rlang_1.1.6            
## [137] htmlwidgets_1.6.4       shiny_1.8.0             farver_2.1.2            jquerylib_0.1.4        
## [141] jsonlite_2.0.0          statnet.common_4.12.0   RCurl_1.98-1.14         Formula_1.2-5          
## [145] GenomeInfoDbData_1.2.11 ggnetwork_0.5.15        Rcpp_1.0.12             stringi_1.8.3          
## [149] zlibbioc_1.48.0         MASS_7.3-60.0.1         plyr_1.8.9              org.Hs.eg.db_3.18.0    
## [153] pkgbuild_1.4.3          parallel_4.3.2          ggrepel_0.9.5           Biostrings_2.70.1      
## [157] splines_4.3.2           hms_1.1.4               ps_1.9.1                Boruta_9.0.0           
## [161] uuid_1.2-2              igraph_2.2.1            ggpubr_0.6.0            ranger_0.18.0          
## [165] fastcluster_1.3.0       ggsignif_0.6.4          rngtools_1.5.2          reshape2_1.4.4         
## [169] pkgload_1.4.1           XML_3.99-0.19           evaluate_1.0.5          BiocManager_1.30.22    
## [173] nloptr_2.2.1            tzdb_0.5.0              foreach_1.5.2           missForest_1.6.1       
## [177] httpuv_1.6.13           productplots_0.1.2      broom_1.0.12            xtable_1.8-4           
## [181] rstatix_0.7.3           later_1.3.2             ragg_1.5.0              viridisLite_0.4.2      
## [185] memoise_2.0.1           AnnotationDbi_1.64.1    cluster_2.1.6           timechange_0.3.0
```
