# Supplementary material for: MeTime: an R package for reproducible longitudinal metabolomics data analysis
Source: Bioinform Adv. 2026 Aug 31;6(1):vbag252. doi: 10.1093/bioadv/vbag252 (PMC13568824; doi:10.1093/bioadv/vbag252)
Supplement: Supplementary file 1 [file vbag252_supplementary_data.zip › Supplementary-Information.pdf]

Supplementary Information: MeTime: An R package for reproducible longitudinal metabolomics data analysis

Marella, B., Weinisch, P., Vehovec, L., Tran, V., Bless, J. J., Njipouombe Nsangou, Y. A., Kastenmüller, G. & Arnold, M.

2026-08-01

This R Markdown document provides an overview of the classes in **MeTime** and a consolidated set of examples that cover the core analysis pipelines showcased in the **Quickstart** and **Building Pipelines** sections of the package documentation on GitHub. The goal is to demonstrate how a variety of analysis modules can be built within a single, reproducible report while also generating individual reports for each analysis. The **HuMet** data, available with the package, are used as the example dataset.

The HuMet Repository (Weinisch et al., 2024) is a time-resolved, longitudinal human metabolomics data resource designed to characterise normal dynamic metabolic responses to controlled acute physiological challenges. Human metabolism alternates between anabolic and catabolic states in response to food intake, fasting, exercise, cold exposure, and other stressors; these transitions affect numerous metabolic pathways simultaneously. The HuMet data captures these responses by integrating metabolomics measurements from blood, urine, and breath collected from 15 young, healthy male participants undergoing oral glucose and lipid loads, mixed meals, 36-hour fasting, exercise, and cold-stress interventions.

The HuMet Repository includes measurements for samples taken at 56 time points for each subject and integrates metabolite abundances acquired across multiple analytical platforms. It was developed to support exploration of challenge-specific metabolic trajectories, comparisons of systemic responses across interventions, and assessment of complementarity between metabolomics platforms. The assay information is obtained from here and the sample information is obtained from here.

For reproducibility and computational convenience, MeTime distributes a processed HuMet example object rather than the complete repository. In this supplement, `humet_data` represents the full bundled dataset, whereas `humet_subset_data` is used for several demonstrations to reduce computational requirements. The latter contains a selected subset of metabolites and samples that are fully complete and hence are suitable for illustrating the MeTime workflows.

Note: These code chunks are intended to be run after installing the required packages and their dependencies shown below. They rely on bundled example data `humet_object` shipped with the package. When executed, the report will generate plots and results similar to those described in the package documentation on GitHub.

Disclaimer: The analyses presented in this report are intended solely to demonstrate the workflows and reporting functionality available in 'MeTime'. The bundled HuMet data are used as an illustrative example and the analyses described below were not designed for formal biological inference. For biologically focused applications of MeTime, please refer to the case-study and preprint, please see the script here and the interpretation of the results in this preprint (Marella et al., 2025).

Table of Contents

1. S4 classes in MeTime
2. Load necessary libraries
3. Quickstart example
4. Distribution, distances and correlation
5. Dimensionality reduction (UMAP and t-SNE)
6. Imputation and feature selection
7. Conservation index
8. Clustering and eigendata
9. Partial correlation analysis
10. Regression analysis
11. Interoperability: MultiAssayExperiment and maplet
12. Interoperability: Importing a SummarizedExperiment
13. References
14. System information

S4 classes in MeTime

`MeTime` uses two S4 classes to organise data and results, as described below.

metime_analyser

This is the central class in `MeTime`, which stores everything related to the analysis in a single object: the raw and processed datasets (`list_of_data` slot), their row and column metadata (`list_of_row_data` and `list_of_col_data` slots), annotations, and the results generated at each step, and thereby maintains the context of the analysis. An annotation slot is used to define non-metabolomics datasets present in the analyser object such as medication intake. `MeTime` automatically validates the integrity of this object after each pipeline step, so users do not have to worry about breaking the structure. Many functions, such as plotting or printing summaries, operate directly on `metime_analyser`, enabling the user to perform sequential transformations and calculations while always preserving provenance.

meta_results

For meta-analysing results across analyses, `MeTime` uses a second, simpler class called `meta_results`. This class is just a list with a single slot that holds named bundles of comparative outputs, such as plot data, information fields (e.g., the type of calculation and its settings), and plot objects. Functions in the `meta_*` family perform integrative comparisons, including conservation-index comparisons, matrix similarities, network overlaps, feature overlaps, and regression meta-analyses. These functions can be applied to one or two `metime_analyser` objects and return a `meta_results` object.

By separating the core analysis container (`metime_analyser`) from the higher-level meta-analyses container (`meta_results`), `MeTime` keeps the workflow clean. The first class accumulates dataset-centric processing and primary model outputs, while the second summarises cross-analysis comparisons. This design helps to keep the pipeline organised and supports reproducible reporting of both primary and meta-level findings within the same package environment.

Load necessary libraries

Run the following chunk to first install all the necessary packages and load them before continuing with the analysis.

```r
if (!requireNamespace("BiocManager", quietly = TRUE)) {
  install.packages("BiocManager", repos = "https://cloud.r-project.org")
}
if (!requireNamespace("devtools", quietly = TRUE)) {
  install.packages("devtools", repos = "https://cloud.r-project.org")
}

# Use both CRAN + Bioconductor repositories during dependency resolution
options(repos = BiocManager::repositories())

if (!requireNamespace("MeTime", quietly = TRUE)) {
  # Install MeTime and dependencies
  # (set dependencies = TRUE to install Imports/Suggests)
  devtools::install_github("compneurobio/MeTime", dependencies = TRUE)
}

if (!requireNamespace("maplet", quietly = TRUE)) {
  devtools::install_github(repo="krumsieklab/maplet", subdir="maplet")
}

# For smooth rendering of pdf
if(!requireNamespace("tinytex", quietly=T)) {
  install.packages("tinytex")
}

# Update tinytex for rendering purposes
tinytex::install_tinytex()

if(!requireNamespace("MultiAssayExperiment", quietly=T)) {
  install.packages("MultiAssayExperiment")
}

library(MeTime)
library(maplet)
library(SummarizedExperiment)
library(MultiAssayExperiment)
library(S4Vectors)
```

Quickstart example

The quickstart guide demonstrates a minimal pipeline for dimensionality reduction using the `humet_object` example data. We scale the data, convert selected metadata fields to factors, compute a principal component analysis (PCA), and then generate an interactive plot.

Rationale for PCA: Metabolomics datasets like **HuMet** contain many metabolites measured per sample, making direct visualisation challenging. PCA can be used to project the high-dimensional data into a smaller number of orthogonal components that capture the majority of variation. By visualising samples in a two-dimensional space, we can explore whether samples cluster according to e.g. subject, challenge type or time point, identify patterns across subjects, and detect potential outliers that may warrant further investigation.

```
# load example data
data("humet_object")

# choose the dataset within the analyser
which_data <- "humet_subset_data"

# run a minimal PCA workflow
pca_example <- humet_object %>%
  mod_trans_zscore(which_data = which_data) %>%
  mod_mutate(which_data = which_data, type = "row_data",
             Factor.Value.Challenge.Day. = factor(Factor.Value.Challenge.Day.)) %>%
  calc_dimensionality_reduction_samples(
    which_data = which_data,
    type = "PCA",
    cols_for_meta = c("Factor.Value.Challenge.", "Factor.Value.Challenge.Day."),
    name = "PCA_samples"
  ) %>%
  mod_generate_plots(type = "PCA", .interactive = TRUE)

# Generate results report for sharing
pca_example %>% write_report(title="PCA results", file="pca_example.html")
```

Results: The PCA plot shows directional shifts in the group centroids between challenges. For instance, samples of OGTT and PAT tend towards lower PC1 values, SLD towards higher PC1/PC2 values, and Fasting/Stress towards lower PC2 values.

Interpretation: Challenge-related metabolic effects are present, however, there is not a fully discrete separation of challenge groups. This could be due to effects being weakened by inter-individual variation, technical variation, or multiple biological processes which together may outweigh any single challenge-specific pattern in the two-dimensional projection.

Outputs: The results of this analysis along with interactive plots are written to pca_example.html

Distribution, distances and correlation

The following code illustrates how to compute sample and metabolite distributions, correlations and distances. These functions help summarise the characteristics of a dataset and assess similarity between samples and features.

Rationale: To understand which research questions can be addressed with a dataset like **HuMet**, it is important to summarise the general properties of the data. Distribution plots reveal the range and potential skewness of phenotypic or molecular features across samples, while pairwise correlations and distance matrices quantify how similar features or samples are to each other. For the correlation and distance examples, the analysis is restricted arbitrarily to the 72 available fasting observations collected from the first 5 timepoints (t1-t5). This subset provides repeated measurements within a relatively consistent experimental condition (fasting), allowing the workflow to demonstrate metabolite–metabolite correlations as well as between-sample distances while ignoring other challenge conditions.

```
# data distributions
dist_obj <- humet_object %>%
  mod_trans_zscore(which_data=which_data) %>%
  calc_distribution_samples(which_data=which_data,
      cols=c("Factor.Value.Challenge.", "Factor.Value.Identifier."),
      stratifications=NULL,
      name="samples_distribution") %>%
  mod_generate_plots(type="distribution_samples", .interactive=TRUE) %>%
  calc_distribution_metabs(which_data=which_data,
      cols=c("super_pathway", "sub_pathway"),
      name="metabs_distribution") %>%
  mod_generate_plots(type="distribution_metabs", .interactive=TRUE)

# correlations and distances
distances_obj <- humet_object %>%
  mod_trans_zscore(which_data=which_data) %>%
  calc_col_stats(which_data=which_data, cols_for_meta=NULL) %>%
  mod_filter(which_data=which_data, type="row_data", Factor.Value.Challenge. %in% "Fasting",
             time %in% c("t1", "t2", "t3", "t4", "t5")) %>%
  calc_correlation_features(which_data=which_data, method="spearman",
      stratifications=NULL) %>%
  mod_generate_plots(type="pairwise_correlation", .interactive=TRUE) %>%
  calc_distance_samples(which_data=which_data, method="euclidean",
      stratifications=NULL) %>%
  mod_generate_plots(type="pairwise_distance", .interactive=TRUE)


# Generate results report for sharing
distances_obj %>% write_report(title="Distance and correlation",
                                file="distances_correlations.html")

dist_obj %>% write_report(title="Data distribution",
                                file="distributions.html")
```

Results: Sample numbers in the dataset are unevenly distributed across challenge types: OGTT (179 samples), OLTT (165), SLD (164), Fasting (132), PAT (104), and Stress (89). Most of the availble metabolites are lipids (123 metabolites) followed by amino acids (103), together comprising 226/312 metabolites (72%). Within the selected t1–t5 fasting interval, a clear correlation pattern is visible: although the median pairwise Spearman correlation was only 0.07, almost 2000 metabolite pairs had an absolute correlation greater than

0.5. Repeated samples from the same participant were also more similar to each other than samples from different participants, with median Euclidean distances of approximately 11.5 and 21.5, respectively.

Interpretation: The majority of the metabolites covered in this dataset are lipids and amino acida, thereby the resulting biological interpretations are also likely to emphasize these pathways. Within the selected fasting interval, most metabolite pairs showed limited correlations, whereas a smaller subset showed stronger pairwise relationships. The lower within-subject distances illustrate that repeated measurements from the same participant tend to be more similar than measurements from different participants. In longitudinal association analyses, this dependence is usually accounted through participant-level random effects.

Outputs: The distribution summaries are written to distributions.html, while the pairwise correlation and sample-distance results are written to distances_correlations.html with interactive plots for subsequent inspection and sharing.

Dimensionality reduction (UMAP and t-SNE)

Beyond PCA, `MeTime` supports UMAP and t-SNE embeddings. The workflow is identical: prepare the data, choose a dimensionality reduction method and generate plots.

Rationale: Non-linear embedding methods can reveal local sample relationships that may not be apparent in a linear projection. UMAP and t-SNE can be used to represent high-dimensional samples in two dimensions, with both methods preserving local neighbourhood relationships. These visualisations can be used to explore whether nearby samples share participant, challenge, or time-point annotations and to identify potential local clusters or outliers.

```
dimred_obj <- humet_object %>%
  mod_trans_zscore(which_data=which_data) %>%
  mod_mutate(which_data = which_data, type = "row_data",
             Factor.Value.Challenge.Day. = factor(Factor.Value.Challenge.Day.)) %>%
  calc_dimensionality_reduction_samples(which_data=which_data, type="UMAP",
      cols_for_meta=c("Factor.Value.Challenge.", "Factor.Value.Challenge.Day."),
      name="UMAP_samples") %>%
  mod_generate_plots(type="UMAP", .interactive=TRUE) %>%
  calc_dimensionality_reduction_samples(which_data=which_data, type="tSNE",
      cols_for_meta=c("Factor.Value.Challenge.", "Factor.Value.Challenge.Day."),
      name="tSNE_samples") %>%
  mod_generate_plots(type="tSNE", .interactive=TRUE)

# Generate results report for sharing
dimred_obj %>% write_report(title="Dimensionality reduction results", file="dim_red.html")
```

Results: Both the methods cluster samples from the same participant together in a local neighbourhood.

Interpretation: This result complements the result of PCA analysis: UMAP and tSNE do not separate samples based on challenge but rather based on subject.

Outputs: The UMAP and tSNE results along with interactive plots are written to dim_red.html

Imputation and feature selection

MeTime includes utilities for handling missing values and selecting informative features. The following code demonstrates how to filter samples and features based on missingness, perform random forest-based imputation (Stekhoven & Bühlmann, 2012), and set up a dummy medication dataset for feature selection using boruta (Kursa & Rudnicki, 2010).

Rationale: Metabolomics datasets often contain missing measurements due to technical limitations. Filtering out samples and features with considerable missingness improves data quality but reduces power, while random forest imputation leverages correlations among metabolites to infer missing values without imposing strong linear assumptions. Feature selection, such as Boruta, helps identify the most informative features (such as medication intake in this example) that affect metabolite variation across subjects or time points, enabling more focused downstream analyses.

```
which_data_full <- "humet_data"

# remove samples and features with >30% missingness and impute
imputed_object <- humet_object %>%
  mod_filter_samples_by_missingness(threshold=0.3,
                                    which_data=which_data_full) %>%
  mod_filter_features_by_missingness(which_data=which_data_full,
                                     threshold=0.3) %>%
  mod_impute(method="rf", which_data=which_data_full)

# create a dummy medication dataset for demonstration purposes
data_mat <- get_data(humet_object, which_data=which_data)
n_samples <- nrow(data_mat)
n_meds <- 50
sample_ids <- rownames(data_mat)
med_ids <- sprintf("MED_%03d", 1:n_meds)
set.seed(1)
med_data <- matrix(rbinom(n_samples * n_meds, size = 1, prob = 0.2),
                   nrow = n_samples,
                   ncol = n_meds,
                   dimnames = list(sample_ids, med_ids)) %>%
  as.data.frame()

disease_categories <- c(
  "Hypertension", "Diabetes", "Hyperlipidemia",
  "Depression", "Pain", "Cardiovascular disease",
  "Inflammation", "Asthma", "Alzheimer's disease",
  "Parkinson's disease", "Anxiety", "Infection"
)

med_metadata <- data.frame(
  ids = med_ids,
  pathway_or_disease = sample(disease_categories, n_meds, replace = TRUE),
  stringsAsFactors = FALSE
)

# append medication dataset to the analyser (as 'medication_data')
feature_selection_obj <- humet_object %>%
  add_dataset(data=med_data,
              col_data=med_metadata,
              row_data=get_rowdata(humet_object, which_data=which_data),
```

```
              name="medication_data") %>%
  mod_trans_zscore(which_data=which_data)

# Feature selection via Boruta can be computationally expensive and hence commented out.
# The user can run the below code chunk separately if they wish to see these results
# However, we do recommend to perform this analysis on a high-computing cluster for
# better performance

#feature_selection_obj <- feature_selection_obj %>%
#   calc_featureselection_boruta(which_x="medication_data",
#       which_y=which_data,
#       verbose=TRUE,
#       name=paste0(which_data, "_selected_full"),
#       cols_for_meta_x=NULL,
#       cols_for_meta_y=NULL,
#       save_per_run=TRUE,
#       num_cores=4)

saveRDS(imputed_object, "imputed_object_humet.rds")
```

Results: The workflow applied a 30% missingness threshold to samples and metabolites in the full HuMet dataset and then performed random-forest imputation on the remaining missing values. To demonstrate the feature-selection workflow, we generated a synthetic medication dataset containing 50 binary medication variables, each with an expected prevalence of 20%, and attached disease-category annotations to these variables. The Boruta feature-selection calculation is not executed during routine vignette rendering because repeated random-forest fitting can be computationally intensive, particularly when applied across a large number of metabolites.

Note: The synthetic medication variables are generated independently of the **HuMet** dataset and therefore do not represent real medication intake of the participants. However, this analysis is included to demonstrate the structure and use of the Boruta interface rather than to identify medication-related metabolic effects. Confirmed features from Boruta would represent candidate predictors for further investigation, whereas tentative or rejected features would indicate insufficient evidence of reproducible predictive relevance.

Outputs: The imputed dataset is stored in the imputed_object_humet.rds file and there are no files generated for feature selection as the analysis itself is computationally expensive.

Conservation index

The conservation index assesses temporal stability at the subject (metabotype) and metabolite level. Here we compute conservation indices across two example time points (t1 and t2) in the HuMet data.

Rationale: Longitudinal metabolomics studies aim to determine whether metabolic profiles remain stable or change in response to interventions or over time. The conservation index quantifies the similarity of a subject's metabotype across time points and the temporal stabolity of each metabolite across subjects (Yousri et al., 2018). By evaluating conservation between t1 and t2 in the **HuMet** dataset, we can identify metabolites or subjects that exhibit high stability versus those showing significant changes in the given timeframe.

```
ci_obj <- humet_object %>%
  mod_trans_zscore(which_data=which_data) %>%
  mod_mutate(which_data = which_data, type = "row_data",
        Factor.Value.Challenge.Day. = factor(Factor.Value.Challenge.Day.)) %>%
  calc_conservation_metabotype(which_data=which_data,
      stratifications=list(time=c("t1","t2")), verbose=FALSE,
      cols_for_meta=c("Factor.Value.Challenge.", "Factor.Value.Challenge.Day."),
      name="CI_metabotype") %>%
  mod_generate_plots(type="CI_metabotype", .interactive=TRUE) %>%
  calc_conservation_metabolite(which_data=which_data,
      stratifications=list(time=c("t1","t2")), verbose=FALSE,
      cols_for_meta=list(humet_subset_data=c(id="id",
                                             sub_pathway="sub_pathway")),
      name="CI_metabolite") %>%
  mod_generate_plots(type="CI_metabolite", .interactive=TRUE)

# visualize the metabotype conservation plot in report
ci_obj %>% write_report(title="Conservation index results",
                        file="conservation_index.html")
```

Results: Subject-level conservation between t1 and t2 was maximal on the reported conservation-index scale for all 15 participants, although the underlying profile correlations ranged from approximately 0.65 to 0.90, with a median of approximately 0.82. At the metabolite level, 209 of 312 metabolites had a conservation index of 1, and 274 had a conservation index greater than 0.95. Lower conservation values were observed for selected metabolites assigned to acylcarnitine, fatty-acid amide, TCA-cycle, and amino-acid-related pathways.

Interpretation: These results demonstrate strong relative participant-level stability between the two selected fasting time points. A subject-level conservation index of 1 should not be interpreted as indicating identical measurements, because the underlying correlations still vary among participants. Metabolites with lower conservation values show reduced relative stability between t1 and t2.

Outputs: The analysis results along with the interactive plots are written to conservation_index.html

Clustering and eigendata

This section shows how to compute WGCNA-based clusters on the **HuMet** dataset.

Rationale: Groups of metabolites often exhibit coordinated behaviour because they participate in common biochemical pathways. Weighted Gene Co-expression Network Analysis (WGCNA) (Langfelder & Hovarth, 2008) groups metabolites according to similarities in their abundance patterns and represents each detected module using an eigengene, defined as the first principal component of the module. For this illustrative example, all 72 available observations from t1–t5 are used instead of restricting the analysis to the 15 original t1 samples as the current implementation is to detect modules cross-sectionally. Each subject–time-point combination is relabelled as a unique pseudo-subject and assigned to an illustrative t1 baseline, thereby providing a larger cross-sectional matrix for demonstrating module assignment and eigendata generation. This relabelling is performed solely to showcase the MeTime workflow.

```
# Create an illustrative WGCNA object containing the first five time points.
# Repeated observations are relabelled as pseudo-subjects and set to t1 only
# to demonstrate the workflow; this object must not be used for inference.
wgcna_demo_obj <- humet_object %>%
  mod_filter(
    which_data = which_data,
    type = "row_data",
    time %in% paste0("t", 1:5)
  )

# Extract the filtered data and its sample metadata.
wgcna_data <- get_data(wgcna_demo_obj, which_data = which_data)
wgcna_row_data <- get_rowdata(wgcna_demo_obj, which_data = which_data)

old_ids <- rownames(wgcna_data)

# Parse IDs of the form S<subject>_t<time>, for example S1_t3.
subject_number <- as.integer(
  sub("^S([0-9]+)_t[0-9]+$", "\\1", old_ids)
)
time_number <- as.integer(
  sub("^S[0-9]+_t([0-9]+)$", "\\1", old_ids)
)

if (anyNA(subject_number) || anyNA(time_number)) {
  stop("Expected sample IDs in the form S<subject>_t<time>, e.g. S1_t1.")
}

# Derive the size of each subject block (15 for the bundled HuMet data).
n_subjects <- max(subject_number)

# Offset each later time point by one block of subjects:
# S1_t1 -> S1_t1, S1_t2 -> S16_t1, S1_t3 -> S31_t1, etc.
new_subject_number <- subject_number + (time_number - 1L) * n_subjects
new_subject <- paste0("S", new_subject_number)
new_ids <- paste0(new_subject, "_t1")

if (anyDuplicated(new_ids)) {
  stop("Pseudo-subject relabelling produced duplicate sample IDs.")
}
```

```r
# Align row metadata to the data before changing identifiers.
row_match <- match(old_ids, wgcna_row_data$id)
if (anyNA(row_match)) {
  stop("Some data row names could not be matched to row_data$id.")
}
wgcna_row_data <- wgcna_row_data[row_match, , drop = FALSE]

# Preserve the genuine longitudinal identifiers for traceability.
wgcna_row_data$original_id <- wgcna_row_data$id
wgcna_row_data$original_subject <- wgcna_row_data$subject
wgcna_row_data$original_time <- wgcna_row_data$time

# Assign a unique pseudo-subject to every subject-time combination and
# place every selected sample at the pseudo-baseline used by WGCNA.
wgcna_row_data$id <- new_ids
wgcna_row_data$subject <- new_subject
wgcna_row_data$time <- "t1"
rownames(wgcna_row_data) <- new_ids
rownames(wgcna_data) <- new_ids

# Replace only the illustrative dataset inside the copied analyser object.
wgcna_demo_obj@list_of_data[[which_data]] <- wgcna_data
wgcna_demo_obj@list_of_row_data[[which_data]] <- wgcna_row_data

# Confirm that data row names and row metadata remain internally consistent.
stopifnot(
  validate_metime_analyser(
    wgcna_demo_obj,
    which_data = which_data
  )$ok
)

# Eigendata transformation using all relabelled pseudo-baseline samples.
eig_obj <- wgcna_demo_obj %>%
  mod_trans_zscore(which_data = which_data) %>%
  mod_trans_eigendata(
    which_data = which_data,
    append = TRUE,
    cols_for_meta = list(
      humet_subset_data = c(id = "id", super_pathway = "super_pathway")
    ),
    soft_power=2,
    baseline = "t1",
    minClusterSize = 2,
    deepSplit = 3,
    pamRespectsDendro = TRUE
  )

# WGCNA cluster assignment using the same relabelled input.
cluster_obj <- wgcna_demo_obj %>%
  mod_trans_zscore(which_data = which_data) %>%
  calc_clusters_wgcna(
    which_data = which_data,
```

```
    baseline = "t1",
    cols_for_meta = list(
      humet_subset_data = c(id = "id", super_pathway = "super_pathway")
    ),
    soft_power=2,
    minClusterSize = 2,
    deepSplit = 3,
    pamRespectsDendro = TRUE
  )

saveRDS(cluster_obj, "humet_clusters.rds")
```

Results: All 312 metabolites were assigned to 53 modules, labelled 1–53, and no metabolites were assigned to the unclassified module 0. Module sizes ranged from 2 to 90 metabolites, with a median module size of 3. The three largest modules contained 90, 17 and 13 metabolites, respectively, while 18 modules contained only two metabolites.

Interpretation: This analysis demonstrates how MeTime can perform WGCNA-based module assignment, leveraging the correlation structure in the metabolomics data, and generate eigendata from a user-defined sample subset. The large number of small modules is consistent with the deliberately permissive settings, particularly a minimum module size of 2 and a deepSplit value of 3, which favour fine partitioning of the metabolite correlation structure.

Outputs: The WGCNA analysis results are stored in the humet_clusters.rds file.

Partial correlation analysis

Network-based methods can be used to estimate conditional statistical associations among metabolites. Here, a longitudinal Gaussian graphical model is constructed using the early fasting measurements collected at t1–t5.

Rationale: Pairwise Spearman correlations identify associations between metabolites but cannot distinguish direct relationships from those mediated by other variables. Partial correlation networks, constructed using Gaussian graphical models (GGM), estimate conditional dependencies among metabolites while controlling for other covariates such as challenge type (Krumsiek et al., 2011). For this demonstration, the analysis is restricted to fasting observations from t1–t5 and further limited to the 13 participants with complete measurements at all five time points, resulting in a balanced subset of 65 repeated samples. Challenge type is included only to demonstrate the `covariates` argument; because all samples are fasting, it provides no adjustment here.

```
network_obj <- humet_object %>%
  mod_merge_row_data_and_data(which_data=which_data,
        cols_list=list(data=NULL, row_data=c("Factor.Value.Challenge.")),
        name="ggm_data", append=T) %>%
  calc_ggm_genenet(which_data = "ggm_data", threshold = "li", all=FALSE,
                   cols_for_meta = list(humet_subset_data=
                                             c(super_pathway="super_pathway")),
                   covariates = c("Factor.Value.Challenge."),
                   stratifications=list(time=c("t1", "t2", "t3", "t4", "t5")),
                   name="genenet_ggm_results") %>%
    mod_generate_plots(type="network")

# visualise the network in report
network_obj %>% write_report(title="Humet metabolite network", file="networks.html")
```

Results: The resulting network contained 140 connected metabolites and 154 retained edges after multiple testing correction, while 172 metabolites had no retained connections. Of the retained edges, 116 connected metabolites assigned to the same super-pathway and 38 connected metabolites assigned to different super-pathways. Lipids formed the largest represented group, containing 66 metabolites and 90 within-super-pathway edges, followed by amino acids, with 38 metabolites and 18 within-super-pathway edges. The network comprised 32 connected components, of which the largest contained 35 metabolites.

Interpretation: The GGM and pairwise Spearman correlation analyses both use the t1–t5 fasting interval, although the GGM uses the smaller subset of participants with complete observations at all five time points. As described before there are around 2000 metabolite pairs that exhibit strong correlations, while the GGM highlights those pairs with significant correlations after accounting for the remaining metabolites and the longitudinal structure.

Outputs: The analysis results along with the interactive network are written to networks.html

Regression analysis

Finally, we demonstrate a simple linear mixed-effects model using the HuMet data. More complex regression models (e.g. GAMMs) with multiple fixed effects and random effects can be constructed following the same pattern, as outlined in the `MeTime` case studies on GitHub.

Rationale: Longitudinal metabolomics data contains multiple observations per subject, so standard regression assumptions of independence are violated when using these repeated measuremnts. Linear mixed-effects models simultaneously estimate fixed effects (e.g., the influence of the challenge condition) and random effects (e.g., subject-specific variability), enabling inference on covariate effects while accounting for within-subject correlation (Bates et al., 2015; Kuznetsova et al., 2017). In this example, applying a mixed model to the **HuMet** dataset allows us to assess how prolonged fasting vs oral glucose tolerance test (OGTT) influences metabolite levels across subjects.

```
# prepare design matrix for mixed model: one trait of interest and an intercept
col_data <- get_coldata(humet_object, which_data=which_data)
lmm_design <- col_data %>% select(id) %>% mutate(cov=NA, type="met",
                                                  interaction=NA, random=NA)

# specify the phenotype to model (Factor.Value.Challenge.) as a trait
traits <- data.frame(id="Factor.Value.Challenge.", cov=NA, type="trait",
                     random="subject", interaction=NA)
lmm_design <- plyr::rbind.fill(lmm_design, traits)

# build and run the mixed model
mixed_model_obj <- humet_object %>%
  mod_merge_row_data_and_data(which_data=which_data,
      cols_list=list(data=NULL, row_data=c("Factor.Value.Challenge.")),
      name="lmm_data", append=TRUE) %>%
  mod_filter(which_data="lmm_data", type="data",
      Factor.Value.Challenge. %in% c("Fasting", "OGTT")) %>%
  add_data(which_data="lmm_data", type="col_data", x=lmm_design, id="id") %>%
  calc_lmm(which_data="lmm_data",
           name="simple_mixed_model",
           stratifications=NULL,
           cols_for_meta=list(c(sub_pathway="sub_pathway")),
           num_cores=2) %>% mod_generate_plots(type="regression", .interactive=TRUE)

mixed_model_obj %>% write_report(title="Mixed model results",
                                 file="lmm.html")
```

Results: The mixed-effects analysis compared 132 prolonged fasting and 179 OGTT samples across 312 metabolites, using prolonged fasting as the reference and a subject-specific random intercept. OGTT was associated with 198 metabolites after Bonferroni correction and 255 after FDR correction. Significant results included 77 lipids and 70 amino acids. Acylcarnitines, lysolipids, and metabolites in leucine, isoleucine, and valine metabolism were predominantly decreased, whereas carbohydrates, insulin, and most primary and secondary bile acids were increased.

Interpretation: The pairwise linear mixed model identified widespread metabolite-level differences between prolonged fasting and OGTT while accounting for repeated measurements using a subject-specific random intercept. OGTT was associated with lower levels of several acylcarnitines and fatty-acid-related metabolites, together with higher glucose-, insulin-, and bile-acid-related measures. These associations are consistent with systematic metabolic differences between the two conditions. However, the estimates should be interpreted cautiously because samples collected at different time points were grouped under the same challenge condition, probably inflating effect significance.

Outputs: The analysis results along with the interactive plots are written to lmm.html

Interoperability: MultiAssayExperiment and maplet

To demonstrate interoperability with other Bioconductor containers and analysis frameworks, both the full HuMet dataset (`humet_data`) and the processed example dataset (`humet_subset_data`) are converted to a MultiAssayExperiment. The `humet_subset_data` assay is then extracted as a SummarizedExperiment, processed using Mahalanobis-distance-based outlier detection from the maplet package, and converted back into a metime_analyser object. This workflow illustrates how data can be transferred between MeTime and external Bioconductor-compatible tools while preserving sample and longitudinal metadata.

Because maplet already provides dedicated quality-control functionality, MeTime focuses on methods and workflows specific to downstream longitudinal omics analysis. Interoperability allows users to combine the specialised functionality of both packages without duplicating existing methods.

```
####################################################################################

#    Showcasing interoperability with other metabolomics packages (here with maplet)

#    The goal is to convert a metime_analyser object into a MultiAssayExperiment and
#    using it as an input for accessing maplet functionality. Although a simple
#    SummarizedExperiment is sufficient for this purpose, we use this opportunity to
#    showcase that multiple datasets can also be converted to a MultiAssayExperiment
#    object

#####################################################################################


# choose the full dataset for conversion
which_data <- c("humet_data", "humet_subset_data")

# convert the analyser object to a MultiAssayExperiment without splitting by time
mae_obj <- get_convert_to_mae(humet_object, which_data = which_data,
                        split_by_time = FALSE)

# access the SummarizedExperiment corresponding to the dataset (no time splitting)
se_list <- MultiAssayExperiment::experiments(mae_obj)

# Selecting the subset data for the analysis as these samples and metabolites
# belong to the same tissue; we then extract the SummarizedExperiment
# for the next steps as input into maplet
mlt <- se_list[["humet_subset_data"]]

# Apply Mahalanobis distance based outlier detection from the maplet package
# The returned object is the updated SummarizedExperiment
se_mva <- maplet::mt_pre_outlier_detection_mahalanobis(mlt)
```

```
## 2026-08-01 21:09:49.344758 INFO:mt:flagged 60 mahalanobis outliers
```

```
# convert the processed SummarizedExperiment back into a metime_analyser
mva_analyser <- get_convert_from_se(se_mva, name = "humet_subset_data_mva_updated")

# Return the analyser object
mva_analyser
```

```
## Datasets:
```

```
##  - Dataset: humet_subset_data_mva_updated with 833 samples and 312 metabolites
## Results:
```

```r
# The same dataset can also be added to an existing analyser object
humet_object_updated <- add_dataset(humet_object,
    data = mva_analyser@list_of_data[["humet_subset_data_mva_updated"]],
    col_data = mva_analyser@list_of_col_data[["humet_subset_data_mva_updated"]],
    row_data = mva_analyser@list_of_row_data[["humet_subset_data_mva_updated"]],
    name = "humet_subset_data_mva_updated")


# return the updated analyser
humet_object_updated
```

```
## Datasets:
##  - Dataset: humet_data with 833 samples and 2672 metabolites
##  - Dataset: humet_subset_data with 833 samples and 312 metabolites
##  - Dataset: humet_subset_data_mva_updated with 833 samples and 312 metabolites
## Results:
```

Interoperability: Importing a SummarizedExperiment

In addition to the built-in HuMet data, you can convert any compatible dataset into a `metime_analyser`. To demonstrate this we work with the built-in `airquality` dataset. The `airquality` data contain daily measurements of ozone, solar radiation, wind speed and temperature recorded in New York City during the summer of 1973. Each day represents a time point, highlighting the longitudinal structure without involving biological variables.

We first load the dataset, create the required `id`, `subject`, and `time` variables, convert it to a `metime_analyser`, and then perform a simple principal component analysis (PCA).

```
#####################################################################################

#    Showcasing interoperability with SummarizedExperiment

#    The idea is to show how MeTime can be used for existing SummarizedExperiment
#    objects. We showcase this by using the airquality dataset which is longitudinal
#    in nature. We first convert the data into a SummarizedExperiment and later
#    convert it to metime_analyser and perform a simple PCA using MeTime

#   Load a non-biological dataset with longitudinal structure.
#   The airquality dataset contains daily air quality measurements
#   in New York City in 1973.

#####################################################################################

data("airquality", package = "datasets")
# Remove rows with missing values for a clean example
aq <- na.omit(airquality)

# Construct sample identifiers and metadata
# For demonstration, months are treated as subjects and days as time points.
subject_ids <- paste0("NYC", aq$Month)
time_points <- paste0("t", aq$Day)
col_data <- S4Vectors::DataFrame(
    subject = subject_ids,
    time = time_points,
    id = paste0(subject_ids, "_", time_points)
  )

# Build assay matrix with features as rows and samples as columns
assay_mat <- t(as.matrix(aq[, c("Ozone", "Solar.R", "Wind", "Temp")]))
rownames(assay_mat) <- c("Ozone", "Solar.R", "Wind", "Temp")

# Feature metadata
row_data <- S4Vectors::DataFrame(id = rownames(assay_mat))

# Create the SummarizedExperiment
se_weather <- SummarizedExperiment::SummarizedExperiment(
    assays = list(data = assay_mat),
    colData = col_data,
    rowData = row_data
)

# Ensure column names match the sample ids
```

```r
colnames(se_weather) <- col_data$id

# Convert the SummarizedExperiment to a metime_analyser
# You can also use the get_convert_from_mae if you want to convert from MultiAssayExperiment
analyser_weather <- get_convert_from_se(
  se_weather,
  name = "airquality_data"
)

# Perform a simple PCA on the imported dataset
pca_weather <- analyser_weather %>%
    mod_trans_zscore(which_data = "airquality_data") %>%
    calc_dimensionality_reduction_samples(
      which_data = "airquality_data",
      type = "PCA",
      cols_for_meta = NULL,
      name = "PCA_airquality"
    ) %>%
    mod_generate_plots(type="PCA", .interactive=TRUE)

# Save the PCA report
pca_weather %>% write_report(title="PCA analysis of airquality data", file="PCA_airquality.html")
```

This example demonstrates that `MeTime` can ingest longitudinal datasets from domains outside of biology, provided they can be converted to a compatible `SummarizedExperiment` format with sample identifiers (`id`), subject identifiers (`subject`), and time identifiers (`time`). Once converted to a `metime_analyser`, the same modular pipeline can be applied to explore temporal patterns.

References

Bates, D., Mächler, M., Bolker, B., & Walker, S. (2015). Fitting linear mixed-effects models using lme4. Journal of statistical software, 67, 1-48.

Krumsiek, J., Suhre, K., Illig, T., Adamski, J., & Theis, F. J. (2011). Gaussian graphical modeling reconstructs pathway reactions from high-throughput metabolomics data. BMC systems biology, 5(1), 21.

Kursa, M. B., & Rudnicki, W. R. (2010). Feature selection with the Boruta package. Journal of statistical software, 36, 1-13.

Kuznetsova, A., Brockhoff, P. B., & Christensen, R. H. (2017). lmerTest package: tests in linear mixed effects models. Journal of statistical software, 82, 1-26.

Langfelder, P., & Horvath, S. (2008). WGCNA: an R package for weighted correlation network analysis. BMC bioinformatics, 9(1), 559.

Marella, B., Weinisch, P., Bless, J. J., Risacher, S. L., Blach, C., Karu, N., ... & Alzheimer's Disease Neuroimaging Initiative. (2025). A seven-year longitudinal study of the Alzheimer's disease blood metabolome. medRxiv.

Stekhoven, D. J., & Bühlmann, P. (2012). MissForest—non-parametric missing value imputation for mixed-type data. Bioinformatics, 28(1), 112-118.

Weinisch, P., Raffler, J., Römisch-Margl, W., Arnold, M., Mohney, R. P., Rist, M. J., ... & Kastenmüller, G. (2024). The HuMet Repository: Watching human metabolism at work. Cell reports, 43(8).

Yousri, N. A., Kastenmüller, G., Gieger, C., Shin, S. Y., Erte, I., Menni, C., ... & Suhre, K. (2014). Long term conservation of human metabolic phenotypes and link to heritability. Metabolomics, 10(5), 1005-1017.

System Information

```r
Sys.info()[c(1:3,5)]
```

```
##       sysname       release       version       machine
##     "Windows"      "10 x64" "build 19045"      "x86-64"
```

```r
sessionInfo()
```

```
## R version 4.3.2 (2023-10-31 ucrt)
## Platform: x86_64-w64-mingw32/x64 (64-bit)
## Running under: Windows 10 x64 (build 19045)
##
## Matrix products: default
##
##
## locale:
## [1] LC_COLLATE=English_India.utf8  LC_CTYPE=English_India.utf8    LC_MONETARY=English_India.utf8
## [4] LC_NUMERIC=C                   LC_TIME=English_India.utf8
##
## time zone: Europe/Berlin
## tzcode source: internal
##
## attached base packages:
## [1] stats4    stats     graphics  grDevices utils     datasets  methods   base
##
## other attached packages:
##  [1] MeTime_1.0.0                lmerTest_3.2-0              lme4_1.1-38                 Matrix_1.6-5
##  [5] MultiAssayExperiment_1.28.0 maplet_1.1.2                SummarizedExperiment_1.32.0 Biobase_2.62
##  [9] GenomicRanges_1.54.1        GenomeInfoDb_1.38.2         IRanges_2.36.0              S4Vectors_0
## [13] BiocGenerics_0.48.1         MatrixGenerics_1.14.0       matrixStats_1.5.0           magrittr_2.0
## [17] lubridate_1.9.4             forcats_1.0.1               stringr_1.6.0               dplyr_1.1.4
## [21] purrr_1.1.0                 readr_2.1.6                 tidyr_1.3.2                 tibble_3.3.
## [25] ggplot2_4.0.1               tidyverse_2.0.0
##
## loaded via a namespace (and not attached):
##   [1] fs_1.6.6                bitops_1.0-7            devtools_2.4.5          httr_1.4.7
##   [5] RColorBrewer_1.1-3      doParallel_1.0.17       Rgraphviz_2.46.0        numDeriv_2016.8-1.1
##   [9] dynamicTreeCut_1.63-1   profvis_0.3.8           tools_4.3.2             doRNG_1.8.6.2
##  [13] backports_1.5.0         R6_2.6.1                DT_0.34.0               lazyeval_0.2.2
##  [17] uwot_0.1.16             mgcv_1.9-1              urlchecker_1.0.1        withr_3.0.2
##  [21] gridExtra_2.3           tictoc_1.2.1            preprocessCore_1.64.0   fdrtool_1.2.18
##  [25] textshaping_1.0.4       WGCNA_1.73              cli_3.6.5               logging_0.10-111
##  [29] network_1.19.0          labeling_0.4.3          sass_0.4.10             KEGGgraph_1.62.0
##  [33] S7_0.2.1                randomForest_4.7-1.2    systemfonts_1.3.1       bsplus_0.1.5
##  [37] GeneNet_1.2.17          foreign_0.8-86          longitudinal_1.1.13     sessioninfo_1.2.2
##  [41] limma_3.58.1            itertools_0.1-3         readxl_1.4.5            rstudioapi_0.18.0
##  [45] impute_1.76.0           RSQLite_2.3.4           FNN_1.1.4.1             visNetwork_2.1.4
##  [49] generics_0.1.4          shape_1.4.6.1           crosstalk_1.2.2         car_3.1-3
##  [53] zip_2.3.0               GO.db_3.18.0            abind_1.4-8             pathview_1.47.1
##  [57] lifecycle_1.0.5         yaml_2.3.12             carData_3.0-5           SparseArray_1.2.4
##  [61] Rtsne_0.17              grid_4.3.2              blob_1.3.0              promises_1.2.1
```

```
##  [65] crayon_1.5.3            miniUI_0.1.1.1          lattice_0.22-5          KEGGREST_1.42.0
##  [69] sna_2.8                 pillar_1.11.1           knitr_1.51              boot_1.3-28.1
##  [73] corpcor_1.6.10          codetools_0.2-19        glue_1.8.0              data.table_1.18.2.1
##  [77] remotes_2.4.2.1         vctrs_0.6.5             png_0.1-8               rmdformats_1.0.4
##  [81] Rdpack_2.6.5            cellranger_1.1.0        gtable_0.3.6            cachem_1.0.8
##  [85] xfun_0.56               openxlsx_4.2.5.2        rbibutils_2.4.1         S4Arrays_1.2.1
##  [89] mime_0.12               coda_0.19-4.1           reformulas_0.4.3.1      rsconnect_1.6.1
##  [93] survival_3.8-3          ggmosaic_0.4.0          pheatmap_1.0.12         iterators_1.0.14
##  [97] tinytex_0.60            statmod_1.5.0           ellipsis_0.3.2          nlme_3.1-164
## [101] usethis_2.2.2           bit64_4.6.0-1           bslib_0.10.0            irlba_2.3.5.1
## [105] otel_0.2.0              rpart_4.1.23            colorspace_2.1-2        DBI_1.2.3
## [109] Hmisc_5.2-5             nnet_7.3-19             processx_3.8.6          mnormt_2.1.1
## [113] tidyselect_1.2.1        bit_4.6.0               compiler_4.3.2          MatrixEQTL_2.3
## [117] glmnet_4.1-10           graph_1.80.0            htmlTable_2.4.3         desc_1.4.3
## [121] DelayedArray_0.28.0     plotly_4.12.0           bookdown_0.46           checkmate_2.3.3
## [125] scales_1.4.0            psych_2.3.12            callr_3.7.6             digest_0.6.34
## [129] minqa_1.2.8             rmarkdown_2.30          XVector_0.42.0          htmltools_0.5.8.1
## [133] pkgconfig_2.0.3         base64enc_0.1-3         fastmap_1.1.1           rlang_1.1.6
## [137] htmlwidgets_1.6.4       shiny_1.8.0             farver_2.1.2            jquerylib_0.1.4
## [141] jsonlite_2.0.0          statnet.common_4.12.0   RCurl_1.98-1.14         Formula_1.2-5
## [145] GenomeInfoDbData_1.2.11 ggnetwork_0.5.15        Rcpp_1.0.12             stringi_1.8.3
## [149] zlibbioc_1.48.0         MASS_7.3-60.0.1         plyr_1.8.9              org.Hs.eg.db_3.18.0
## [153] pkgbuild_1.4.3          parallel_4.3.2          ggrepel_0.9.5           Biostrings_2.70.1
## [157] splines_4.3.2           hms_1.1.4               ps_1.9.1                Boruta_9.0.0
## [161] uuid_1.2-2              igraph_2.2.1            ggpubr_0.6.0            ranger_0.18.0
## [165] fastcluster_1.3.0       ggsignif_0.6.4          rngtools_1.5.2          reshape2_1.4.4
## [169] pkgload_1.4.1           XML_3.99-0.19           evaluate_1.0.5          BiocManager_1.30.22
## [173] nloptr_2.2.1            tzdb_0.5.0              foreach_1.5.2           missForest_1.6.1
## [177] httpuv_1.6.13           productplots_0.1.2      broom_1.0.12            xtable_1.8-4
## [181] rstatix_0.7.3           later_1.3.2             ragg_1.5.0              viridisLite_0.4.2
## [185] memoise_2.0.1           AnnotationDbi_1.64.1    cluster_2.1.6           timechange_0.3.0
```
