# Supplementary material for: MeTime: an R package for reproducible longitudinal metabolomics data analysis
Source: Bioinform Adv. 2026 Aug 31;6(1):vbag252. doi: 10.1093/bioadv/vbag252 (PMC13568824; doi:10.1093/bioadv/vbag252)
Supplement: Supplementary file 1 [file vbag252_supplementary_data.zip › lmm.html]

lmm


Code 

- Show All Code
- Hide All Code

# lmm

## lmm

- simple\_mixed\_model
  - Analyzer
    info
  - Plots
- Session
  info

2026-08-01

# simple\_mixed\_model

## Analyzer info

```
## Datasets: 
##  - Dataset: humet_data with 833 samples and 2672 metabolites
##  - Dataset: humet_subset_data with 833 samples and 312 metabolites
##  - Dataset: lmm_data with 311 samples and 313 metabolites
## Results: 
## 1.simple_mixed_model : 
##  -  Linear mixed models for lmm_data_Factor.Value.Challenge.OGTT
```

object %>%   
Copy to Clipboard

mod\_merge\_row\_data\_and\_data(which\_data = 'humet\_subset\_data',mutations = list(data = NULL, row\_data = 'Factor.Value.Challenge.'),name = 'lmm\_data') %>%   
 mod\_filter(which\_data = 'lmm\_data',type = 'data',mutations = 'Factor.Value.Challenge. %in% c('Fasting', 'OGTT')') %>%   
 add\_data(which\_data = 'lmm\_data',x = c('id', 'cov', 'type', 'interaction', 'random'),id = 'id',type = 'col\_data') %>%   
 calc\_lmm(which\_data = 'lmm\_data',verbose = TRUE,cols\_for\_meta = list(c(sub\_pathway = 'sub\_pathway')),num\_cores = 2,threshold = c('none', 'nominal', 'li', 'fdr', 'bonferroni'),name = 'simple\_mixed\_model',stratifications = NULL)

×

#### mod\_merge\_row\_data\_and\_data

Modification (mod) function to merge data and row\_data (sample info)  
partially or completely.  
  
Description:  
  
     a modification function that merges partial or complete data and  
     row\_data of a dataset. If you want to use certain columns for the  
     rest of the analysis properly for annotating plots then use  
     mod\_mutate function to rename the colnames and rownames (if  
     applicable) to maintain consistency in the merged dataset  
  
Usage:  
  
       
         (  
       object,  
       which\_data,  
       cols\_list = list(data = NULL, row\_data = NULL),  
       append = FALSE,  
       name = 'merged\_data'  
     )  
       
Arguments:  
  
  object: an S4 object of class metime\_analyser.  
  
which\_data: a character defining which data and row\_data should be  
          merged. Has to contain only one value.  
  
cols\_list: A list of named character vectors to merge dataset. example:  
          which\_data = 'dataset1'; cols\_list = list(data=c(...),  
          row\_data=c(...)) or list(c(...), c(...)) If you want full  
          data set cols\_list=list(data=NULL, row\_data=NULL) which is  
          the default setting  
  
    name: a character to define the name of a new dataset.  
  
Value:  
  
     a new S4 object of class metime\_analyser with the new merged  
     dataset appended to it  
  
See Also:  
  
     mod\_merge\_results, mod\_merge\_data

Close

×

#### mod\_filter

Modification function to filter columns in data, row\_data or col\_data  
  
Description:  
  
     Modification (mod) function to filter columns in data, row\_data or  
     col\_data. It is a wrapper function to dplyr::filter() made for  
     metime\_analyser objects.  
  
Usage:  
  
       
         (object, which\_data, ..., type = 'data')  
       
Arguments:  
  
  object: A S4 object of class metime\_analyser.  
  
which\_data: a vector of character defining which data/result is to be  
          filtered.  
  
     ...: arguments to pass directly into dplyr::filter() function.  
  
    type: character input of length 1 to define the type of data to be  
          manipulated. Accepted inputs are 'row\_data', 'col\_data',  
          'data' and 'results'. However renamed results will be  
          returned to the user as a list of results and will not return  
          the full analyser object  
  
Value:  
  
     object with mutated data, col\_data or row\_data. However, if type  
     is 'results' then plot\_data with modifications expected will be  
     returned  
  
See Also:  
  
     mod\_mutate, mod\_rename, mod\_select

Close

×

#### add\_data

Add a data frame to analyzer object.  
  
Description:  
  
     Add a data.frame x to existing data within an analyzer object.  
     Data can be added to data, col\_data or row\_data. A full join of  
     the data is used.  
  
Usage:  
  
       
         (object, which\_data, type, x, id)  
       
Arguments:  
  
  object: a S4 object of the class 'metime\_analyzer'.  
  
which\_data: a character to define which dataset is to be used.  
  
    type: a character defining the data which should be appended. Can  
          only be data, col\_data, row\_data. Default set to data.  
  
       x: a data.frame that is merged to the data.  
  
      id: a character vector defining the column to be used for  
          matching. Default set to NULL (first column of x will be used  
          as id column)  
  
Value:  
  
     a S4 object of class 'metime\_analyser' with appended data.frame x

Close

×

#### calc\_lmm

Calculation of linear mixed models  
  
Description:  
  
     Fits multiple Linear mixed models (LMM) to a longitudinal dataset.  
  
Usage:  
  
       
         (  
       object,  
       which\_data,  
       stratifications = NULL,  
       cols\_for\_meta = NULL,  
       threshold = c('none', 'nominal', 'li', 'fdr', 'bonferroni'),  
       verbose = T,  
       name = 'regression\_lmm\_1',  
       num\_cores = NULL,  
       cluster\_profile = c('auto', 'local', 'hpc'),  
       hpc\_libpaths = NULL  
     )  
       
Arguments:  
  
  object: an S4 object of class metime\_analyser  
  
which\_data: a character defining the name of the dataset to be used.  
  
stratifications: list to stratify data into a subset. Usage  
          list(name=value). Default set to NULL, thereby not performing  
          any type of stratification.  
  
cols\_for\_meta: a list of character vector to define column names that  
          are to be used for plotting purposes. Default set to NULL,  
          therby not adding columns as metadata. If you want automated  
          facet wrapping option then set your new\_columns as  
          'facet\_your\_name'  
  
threshold: a character of length 1 to define the type of threshold for  
          significant interactions. allowed inputs are 'li', 'FDR',  
          'bonferroni' and 'nominal'(cutoff p=0.05, set as Default)  
  
 verbose: a logical on whether to print the calculation progress.  
          Default set to FALSE.  
  
    name: a character vector to define the index within the results.  
          Should be equal to length of which\_data. Default set to  
          regression\_gamm\_1.  
  
num\_cores: numeric input to define the number of cores that you want to  
          use for parallel computing. Default is set to NULL which is  
          parallel::detectCores() -1.  
  
cluster\_profile: Character string controlling worker initialization  
          profile. One of ''auto'', ''local'', or ''hpc''.  
  
            \* ''auto'': detects common scheduler environments (e.g.  
              SLURM/PBS/LSF) and chooses ''hpc'' when detected.  
  
            \* ''local'': standard local machine setup with default  
              library paths.  
  
            \* ''hpc'': cluster-oriented setup; can prepend worker  
              library paths via 'hpc\_libpaths'.  
  
hpc\_libpaths: Optional character vector of library paths to prepend on  
          worker nodes when 'cluster\_profile = 'hpc''. Use this when  
          compute nodes need explicit '.libPaths()' (e.g. non-shared  
          user libraries). Ignored for ''local'' profile.  
  
  random: a character vector defining which variables should be treated  
          as random effects. Default set to 'subject'.  
  
interaction: a character vector defining which interaction terms should  
          be added to the model. Default set to NULL, with no  
          interaction added.  
  
Details:  
  
     Add details here  
  
Value:  
  
     a S4 object of the class metime\_analyzer with analysis results  
     appended to the result section.

Close

## Plots

### Factor.Value.Challenge.OGTT

Linear mixed models for lmm\_data\_Factor.Value.Challenge.OGTT

# Session info

```
print(sessionInfo())
```

```
## R version 4.3.2 (2023-10-31 ucrt)
## Platform: x86_64-w64-mingw32/x64 (64-bit)
## Running under: Windows 10 x64 (build 19045)
## 
## Matrix products: default
## 
## 
## locale:
## [1] LC_COLLATE=English_India.utf8  LC_CTYPE=English_India.utf8    LC_MONETARY=English_India.utf8
## [4] LC_NUMERIC=C                   LC_TIME=English_India.utf8    
## 
## time zone: Europe/Berlin
## tzcode source: internal
## 
## attached base packages:
## [1] stats4    stats     graphics  grDevices utils     datasets  methods   base     
## 
## other attached packages:
##  [1] MeTime_1.0.0                lmerTest_3.2-0              lme4_1.1-38                 Matrix_1.6-5               
##  [5] MultiAssayExperiment_1.28.0 maplet_1.1.2                SummarizedExperiment_1.32.0 Biobase_2.62.0             
##  [9] GenomicRanges_1.54.1        GenomeInfoDb_1.38.2         IRanges_2.36.0              S4Vectors_0.40.2           
## [13] BiocGenerics_0.48.1         MatrixGenerics_1.14.0       matrixStats_1.5.0           magrittr_2.0.3             
## [17] lubridate_1.9.4             forcats_1.0.1               stringr_1.6.0               dplyr_1.1.4                
## [21] purrr_1.1.0                 readr_2.1.6                 tidyr_1.3.2                 tibble_3.3.1               
## [25] ggplot2_4.0.1               tidyverse_2.0.0            
## 
## loaded via a namespace (and not attached):
##   [1] fs_1.6.6                bitops_1.0-7            devtools_2.4.5          httr_1.4.7             
##   [5] RColorBrewer_1.1-3      doParallel_1.0.17       Rgraphviz_2.46.0        numDeriv_2016.8-1.1    
##   [9] dynamicTreeCut_1.63-1   profvis_0.3.8           tools_4.3.2             doRNG_1.8.6.2          
##  [13] backports_1.5.0         R6_2.6.1                DT_0.34.0               lazyeval_0.2.2         
##  [17] uwot_0.1.16             mgcv_1.9-1              urlchecker_1.0.1        withr_3.0.2            
##  [21] gridExtra_2.3           tictoc_1.2.1            preprocessCore_1.64.0   fdrtool_1.2.18         
##  [25] textshaping_1.0.4       WGCNA_1.73              cli_3.6.5               logging_0.10-111       
##  [29] network_1.19.0          labeling_0.4.3          sass_0.4.10             KEGGgraph_1.62.0       
##  [33] S7_0.2.1                randomForest_4.7-1.2    systemfonts_1.3.1       bsplus_0.1.5           
##  [37] GeneNet_1.2.17          foreign_0.8-86          longitudinal_1.1.13     sessioninfo_1.2.2      
##  [41] limma_3.58.1            itertools_0.1-3         readxl_1.4.5            rstudioapi_0.18.0      
##  [45] impute_1.76.0           RSQLite_2.3.4           FNN_1.1.4.1             visNetwork_2.1.4       
##  [49] generics_0.1.4          shape_1.4.6.1           crosstalk_1.2.2         car_3.1-3              
##  [53] zip_2.3.0               GO.db_3.18.0            abind_1.4-8             pathview_1.47.1        
##  [57] lifecycle_1.0.5         yaml_2.3.12             carData_3.0-5           SparseArray_1.2.4      
##  [61] Rtsne_0.17              grid_4.3.2              blob_1.3.0              promises_1.2.1         
##  [65] crayon_1.5.3            miniUI_0.1.1.1          lattice_0.22-5          KEGGREST_1.42.0        
##  [69] sna_2.8                 pillar_1.11.1           knitr_1.51              boot_1.3-28.1          
##  [73] corpcor_1.6.10          codetools_0.2-19        glue_1.8.0              data.table_1.18.2.1    
##  [77] remotes_2.4.2.1         vctrs_0.6.5             png_0.1-8               rmdformats_1.0.4       
##  [81] Rdpack_2.6.5            cellranger_1.1.0        gtable_0.3.6            cachem_1.0.8           
##  [85] xfun_0.56               openxlsx_4.2.5.2        rbibutils_2.4.1         S4Arrays_1.2.1         
##  [89] mime_0.12               coda_0.19-4.1           reformulas_0.4.3.1      rsconnect_1.6.1        
##  [93] survival_3.8-3          ggmosaic_0.4.0          pheatmap_1.0.12         iterators_1.0.14       
##  [97] tinytex_0.60            statmod_1.5.0           ellipsis_0.3.2          nlme_3.1-164           
## [101] usethis_2.2.2           bit64_4.6.0-1           bslib_0.10.0            irlba_2.3.5.1          
## [105] otel_0.2.0              rpart_4.1.23            colorspace_2.1-2        DBI_1.2.3              
## [109] Hmisc_5.2-5             nnet_7.3-19             processx_3.8.6          mnormt_2.1.1           
## [113] tidyselect_1.2.1        bit_4.6.0               compiler_4.3.2          MatrixEQTL_2.3         
## [117] glmnet_4.1-10           graph_1.80.0            htmlTable_2.4.3         desc_1.4.3             
## [121] DelayedArray_0.28.0     plotly_4.12.0           bookdown_0.46           checkmate_2.3.3        
## [125] scales_1.4.0            psych_2.3.12            callr_3.7.6             digest_0.6.34          
## [129] minqa_1.2.8             rmarkdown_2.30          XVector_0.42.0          htmltools_0.5.8.1      
## [133] pkgconfig_2.0.3         base64enc_0.1-3         fastmap_1.1.1           rlang_1.1.6            
## [137] htmlwidgets_1.6.4       shiny_1.8.0             farver_2.1.2            jquerylib_0.1.4        
## [141] jsonlite_2.0.0          statnet.common_4.12.0   RCurl_1.98-1.14         Formula_1.2-5          
## [145] GenomeInfoDbData_1.2.11 ggnetwork_0.5.15        Rcpp_1.0.12             stringi_1.8.3          
## [149] zlibbioc_1.48.0         MASS_7.3-60.0.1         plyr_1.8.9              org.Hs.eg.db_3.18.0    
## [153] pkgbuild_1.4.3          parallel_4.3.2          ggrepel_0.9.5           Biostrings_2.70.1      
## [157] splines_4.3.2           hms_1.1.4               ps_1.9.1                Boruta_9.0.0           
## [161] uuid_1.2-2              igraph_2.2.1            ggpubr_0.6.0            ranger_0.18.0          
## [165] fastcluster_1.3.0       ggsignif_0.6.4          rngtools_1.5.2          reshape2_1.4.4         
## [169] pkgload_1.4.1           XML_3.99-0.19           evaluate_1.0.5          BiocManager_1.30.22    
## [173] nloptr_2.2.1            tzdb_0.5.0              foreach_1.5.2           missForest_1.6.1       
## [177] httpuv_1.6.13           productplots_0.1.2      broom_1.0.12            xtable_1.8-4           
## [181] rstatix_0.7.3           later_1.3.2             ragg_1.5.0              viridisLite_0.4.2      
## [185] memoise_2.0.1           AnnotationDbi_1.64.1    cluster_2.1.6           timechange_0.3.0
```
