# Supplementary material for: MeTime: an R package for reproducible longitudinal metabolomics data analysis
Source: Bioinform Adv. 2026 Aug 31;6(1):vbag252. doi: 10.1093/bioadv/vbag252 (PMC13568824; doi:10.1093/bioadv/vbag252)
Supplement: Supplementary file 1 [file vbag252_supplementary_data.zip › Supplementary Table 1.docx]

**Supplementary Table 1:** MeTime in comparison to complementary existing metabolomics toolboxes/workflows.

| **Framework** | **Main role & data type** | **Difference to MeTime** | **Interoperability with MeTime** |
| --- | --- | --- | --- |
| **MetaboAnalyst / MetaboAnalystR**  **(Chong et al., 2018)** | Uses metabolomics abundance data; offers univariate tests (t‑test, ANOVA), multivariate models, and pathway/enrichment analyses. | Emphasises cross‑sectional & simple univariate/multivariate statistics; lacks complex analysis such as mixed‑effects models or feature selection; limited upload size in web server (~50 MB). | Use MetaboAnalyst for data processing, univariate tests, and functional interpretation; then export data to MeTime to perform more complex analysis. |
| **Maplet**  **(Chetnik et al., 2022)** | R-based modular workflows using metabolite abundance data; broad preprocessing and single‑dataset statistics. | Designed for single datasets; limited support for synchronised multi‑dataset and no longitudinal analysis methods. | Perform preprocessing and exploratory analysis in maplet; transfer data into MeTime for downstream longitudinal analyses. |
| **MeTEor**  **(Grabert et al., 2025)** | Shiny app for exploring longitudinal metabolomics abundance data; accepts abundance data and includes PCA and basic mixed‑model tests | Interactive tool; less scriptable and lacks meta-analysis; ID mapping and batch correction are manual | Use MeTEor for quick visual exploration; then use the same data for scripted longitudinal pipelines, advanced models (e.g., generalised additive) in MeTime |
| **Workflow4Metabolomics (W4M)**  **(Giacomoni et al., 2015)** | Galaxy-based platform for raw spectral data (LC‑MS, GC‑MS, NMR) preprocessing, normalisation, batch correction, and annotation. | Operates on spectral data and focuses on preprocessing; does not support downstream longitudinal data handling or analyses. | Preprocess and annotate raw LC-MS/NMR data in W4M; export abundance data and metadata to MeTime for longitudinal downstream analyses. |
| **Rodin**  **(Minasenko et al., 2025)** | Python/web tool for metabolite abundance data; includes t‑test, ANOVA, PLS‑DA, logistic/linear regression, and random forests. | Focused on cross‑sectional statistics and small uploaded files (≤150 MB); missing values imputed as zero; lacks mixed‑effects models and meta-analysis. | Use Rodin for quick cross‑sectional exploration; then supply cleaned datasets to MeTime for longitudinal analyses and multi‑dataset integration |
| **XCMS**  **(Smith et al., 2006)** | R package for LC‑MS raw-data preprocessing, including chromatographic peak detection, alignment, and feature correspondence | Works on raw spectral files; no downstream longitudinal analysis. | Use XCMS to generate peak tables from raw LC‑MS data; import the quantified abundance data into MeTime for normalisation, imputation, and advanced longitudinal analysis. |

**References:**

Chetnik, K., Benedetti, E., Gomari, D. P., Schweickart, A., Batra, R., Buyukozkan, M., ... & Krumsiek, J. (2022). maplet: an extensible R toolbox for modular and reproducible metabolomics pipelines. *Bioinformatics*, *38*(4), 1168-1170.

Chong, J., Soufan, O., Li, C., Caraus, I., Li, S., Bourque, G., ... & Xia, J. (2018). MetaboAnalyst 4.0: towards more transparent and integrative metabolomics analysis. *Nucleic acids research*, *46*(W1), W486-W494.

Giacomoni, F., Le Corguille, G., Monsoor, M., Landi, M., Pericard, P., Pétéra, M., ... & Caron, C. (2015). Workflow4Metabolomics: a collaborative research infrastructure for computational metabolomics. *Bioinformatics*, *31*(9), 1493-1495.

Grabert, G., Dehncke, D., More, T., List, M., Kraft, A. R., Cornberg, M., ... & Kacprowski, T. (2024). MeTEor: an R Shiny app for exploring longitudinal metabolomics data. *Bioinformatics Advances*, *4*(1), vbae178.

Minasenko, B., Wang, D., Cirillo, P., Krigbaum, N., Cohn, B., Jones, D. P., ... & Hu, X. (2025). Rodin: a streamlined metabolomics data analysis and visualization tool. *Bioinformatics advances*, *5*(1), vbaf088.

Smith, C. A., Want, E. J., O'Maille, G., Abagyan, R., & Siuzdak, G. (2006). XCMS: processing mass spectrometry data for metabolite profiling using nonlinear peak alignment, matching, and identification. *Analytical chemistry*, *78*(3), 779-787.
